# Diversity of SCC*mec* elements and *spa* types in South African *Staphylococcus aureus mec*A-positive blood culture isolates

**DOI:** 10.1186/s12879-020-05547-w

**Published:** 2020-11-10

**Authors:** Ashika Singh-Moodley, Michelle Lowe, Ruth Mogokotleng, Olga Perovic

**Affiliations:** 1 National Institute for Communicable Diseases, a Division of the National Health Laboratory Service, Centre for Healthcare-Associated Infections, Antimicrobial Resistance and Mycoses, 1 Modderfontein Road, Sandringham, Johannesburg, 2131 South Africa; 2 Faculty of Health Sciences, School of Pathology, Department of Clinical Microbiology and Infectious Diseases, University of the Witwatersrand, Private Bag 3, Wits, Johannesburg, 2050 South Africa

**Keywords:** Methicillin-resistant *Staphylococcus aureus*, SCC*mec* typing, *Spa* typing

## Abstract

**Background:**

The prevalence of *Staphylococcus aureus* varies depending on the healthcare facility, region and country. To understand its genetic diversity, transmission, dissemination, epidemiology and evolution in a particular geographical location, it is important to understand the similarities and variations in the population being studied. This can be achieved by using various molecular characterisation techniques. This study aimed to provide detailed molecular characterisation of South African *mec*A-positive *S. aureus* blood culture isolates by describing the SCC*mec* types, *spa* types and to lesser extent, the sequence types obtained from two consecutive national surveillance studies.

**Methods:**

*S. aureus* blood culture isolates from a national laboratory-based and enhanced surveillance programme were identified and antimicrobial susceptibility testing was performed using automated systems. A real-time PCR assay confirmed the presence of the methicillin-resistance determinant, *mec*A. Conventional PCR assays were used to identify the SCC*mec* type and *spa* type, which was subsequently analysed using the Ridom StaphType™ software. Multilocus sequence typing was performed on selected isolates using conventional methods. MRSA clones were defined by their sequence type (ST), SCC*mec* type and *spa* type.

**Results:**

A detailed description of findings is reported in this manuscript. SCC*mec* type III predominated overall followed by type IV. A total of 71 different *spa* types and 24 novel *spa* types were observed. *Spa* type t037 was the most common and predominated throughout followed by t1257. Isolates were multidrug resistant; isolates belonging to all SCC*mec* types were resistant to most of the antibiotics with the exception of type I; isolates with *spa* type t045 showed resistance to all antibiotics except vancomycin. The most diverse SCC*mec*-*spa* type complex was composed of the SCC*mec* type IV element and 53 different *spa* types.

**Conclusion:**

Although ST data was limited, thereby limiting the number of clones that could be identified, the circulating clones were relatively diverse.

## Introduction

*Staphylococcus aureus* bacteraemia is an important cause of morbidity and mortality in both healthcare-associated (HA) and community-associated (CA) infections worldwide [1, 2]. *S. aureus* is responsible for an extensive range of human diseases, including bloodstream infections, pneumonia, endocarditis, food poisoning, toxic shock syndrome, skin and soft tissue infections, and bone and joint infections [3, 4]. The prevalence of *S. aureus* varies depending on the healthcare facility, region and country. Furthermore, the prevalence of methicillin-susceptible *S. aureus* (MSSA) and methicillin-resistant *S. aureus* (MRSA) may also differ. In order to understand the genetic diversity, transmission, dissemination, epidemiology and evolution of MSSA and MRSA clones in a particular geographical location, it is important to acquire knowledge on the similarities and variations in the population being studied. This is not only important for epidemiological surveys but also for infection prevention and control policies [5]. This can be achieved by employing the use of various molecular characterisation techniques [2]. Reliable molecular techniques that have been used for typing *S. aureus* include Pulsed-field Gel Electrophoresis (PFGE), Multilocus Sequence Typing (MLST), Stapylococcal protein A (*spa*) typing and Staphylococcal Cassette Chromosome *mec* (SCC*mec*) typing [2, 6].

PFGE is based on the DNA banding pattern obtained after digesting the bacterial genome with a restriction enzyme [7]. MLST and its clustering algorithm, Based Upon Related Sequence Type (BURST) classifies isolates according to nucleotide variations in seven housekeeping/reference genes (loci) [5]. These genes are sequenced and a unique allele number is assigned using an online programme specific to the MLST scheme. A combination of the allele numbers (i.e. allelic profile) produces a particular sequence type (ST) for a bacterial strain. Those with similar STs are grouped together in a single clonal complex (CC) [6, 8]. *Spa* typing sequences the *S. aureus*-specific staphylococcal protein A (*spa*) gene which is one of the virulence factors on the surface of the organism preventing phagocytosis by the immune system [9]. *Spa* typing and its clustering algorithm, Based Upon Repeat Pattern (BURP) is based on the sequencing of a polymorphic 24 bp region of the *spa* gene. This is a variable-number tandem repeat (VNTR) sequence within the 3′ coding region [4]. The repeat regions are assigned a numerical code and the *spa* type is determined by the order of specific repeats [3]. Studies have shown that *spa* typing produced results that are notably comparable with that of MLST [6, 10]. Due to lower implementation costs and that only a single locus needs to be sequenced, *spa* typing has shown to be more efficient and results are consistent across different settings, specimen type and patient age [6]. Therefore *spa* typing has been shown to be appropriate for use in evolutionary and macro-epidemiology studies [4, 6, 11, 12]. However, as recombination events in a single locus can distort clonal relationships, there is the question of how a method that sequences only a single locus can be used for macro-epidemiology studies [13]. SCC*mec* typing classifies SCC*mec* elements according to their structural differences [5]. It involves the typing of the staphylococcal cassette chromosome *mec,* which is a mobile genetic element and harbours the methicillin-resistance determinant gene. This element is genetically diverse with many types, subtypes and variants being reported [14]. The molecular organisation of the cassette is complex, but it can be broken down into three structural components, which include: i) the cassette chromosome recombinase (*ccr*) gene complex, ii) the *mec* gene complex and iii) the joining (J) regions [15, 16]. The *ccr* gene complex encodes site-specific recombinases for the excision and insertion of the element into the chromosome [14, 16, 17]. This complex therefore affords the SCC*mec* element mobility and thus facilitates its transfer to other staphylococcal species [16]. The *mec* complex confers methicillin resistance as it consists of the *mec* gene, its regulatory genes, the *mec*I and the *mec*R1 genes and various insertion sequences [14, 18]. A combination of both the *ccr* gene complex and the *mec* gene class is used to assign the specific SCC*mec* type. Thirteen SCC*mec* types (I-XIII) have been defined in MRSA based on complete sequence data [17, 19–21]; International Working Group on the Staphylococcal Cassette Chromosome elements (IWG-SCC) (2015) Available online: http://www.sccmec.org).

Although we have previously described the MRSA population in South Africa [22–25], a detailed description of the SCC*mec* types and *spa* types is lacking. This study therefore reports on the various clones present in our MRSA study population by SCC*mec* and *spa* type combinations (SCC*mec*-*spa* type complexes). Moreover, although MLST data was lacking for the majority of our sample population, the predominating circulating clones (ST-SCC*mec*-*spa* type) based on the most common *spa* types were described.

## Materials and methods

### Bacterial strains and phenotypic methods

A case of *S. aureus* bacteraemia was defined as the isolation of *S. aureus* from a blood culture. Blood culture isolates, which formed part of the GERMS-SA laboratory-based and enhanced antimicrobial resistance surveillance studies from sentinel centres in South Africa were submitted and participation was voluntary. The first was a two-year laboratory-based surveillance study (June 2010 to July 2012); sites represented 13 sentinel centres from the Gauteng, KwaZulu-Natal, Free State and Western Cape provinces. The second was an enhanced surveillance study (August 2012 to December 2017); sites represented five sentinel centres from six large academic hospitals from the Gauteng and the Western Cape provinces. A 21-day exclusion period was applied to avoid duplicate isolates of the organism from the same patient.

In total, 5820 viable isolates [MSSA (*n* = 3801) and MRSA (*n* = 2019)] were submitted on Dorset transport media (Diagnostic Media Products (DMP), National Health Laboratory Service (NHLS), Johannesburg, South Africa). Each isolate was plated onto a 5% blood agar plate (DMP, NHLS, Johannesburg, South Africa) followed by organism identification and antimicrobial susceptibility testing using automated systems. Organism identification was done using VITEK® II (bioMèrieux, France) or MALDI-TOF MS (Microflex, Bruker Daltonics, MA, USA) and antimicrobial susceptibility testing (AST) was done using the MicroScan Walkaway system (Gram-positive panel PM33) (Siemens, Sacramento, CA, USA). Interpretation of susceptibility was performed according to the Clinical and Laboratory Standards Institute (CLSI) guidelines [26]. Bacterial cells were lysed at 95 °C for 25 min and the DNA was extracted and used in the genotypic assays.

### Polymerase chain reaction (PCR) screening for *mecA* in MRSA isolates

The LightCycler 480 II (Roche Applied Science) instrument was used for the real-time PCR of *mec*A and *nuc,* which were amplified in a multiplex assay using the LightCycler 480 Probes Master kit (Roche Diagnostics, IN, USA) with previously published primers and probes [27].

### SCC*mec* typing

All 2019 *me*cA-positive MRSA isolates were typed by a multiplex PCR assay using the Qiagen Multiplex PCR kit (Qiagen, Germany) and previously published primers [28].

### *Spa*-typing

*Spa*-typing was performed on 1467 MRSA isolates. The *spa* gene was amplified using previously published primers [12] and the Amplitaq Gold DNA Polymerase kit (Applied Biosystems, CA, USA). Purified PCR products (Qiagen Purification kit; Qiagen, Germany) were sequenced (Inqaba Biotech, South Africa). Sequences were assembled using CLC Bio main workbench (Qiagen, Germany) and analysed using the Ridom StaphType™ software, (Ridom GmbH, Würzburg, Germany).

### Multilocus sequence typing (MLST)

Multilocus Sequence Typing was performed on 48 isolates, which were selected randomly based on the most common *spa*-types. Primers [29] amplifying seven reference genes were used. Amplification was done using the Amplitaq Gold DNA Polymerase kit (Applied Biosystems, CA, USA). Purified PCR products were sequenced (Inqaba Biotech, South Africa). Sequences were assembled using the CLC Bio main workbench (Qiagen, Germany) and analysed using the online database (https://pubmlst.org/saureus/).

## Results

### SCC*mec* typing

**Fig. 1:**
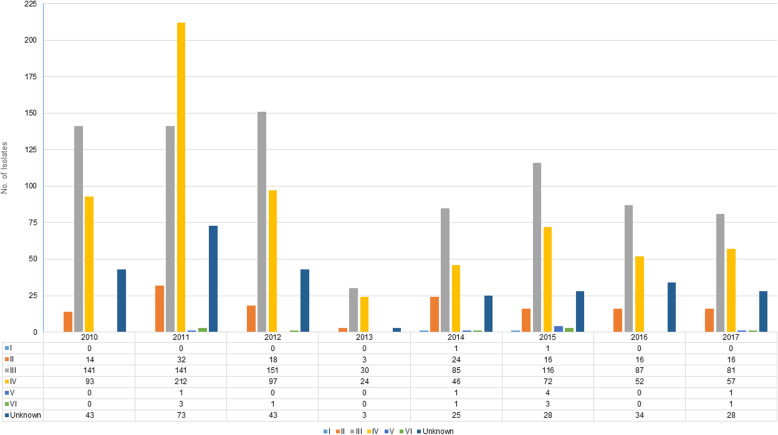
Distribution of SCC*mec* types per year in *mec*A-positive isolates (*n* = 2019)

The distribution of SCC*mec* types per year in 2019 *mec*A-positive isolates is seen in Fig. 1. SCC*mec* type III predominated every year followed by type IV with the exception of 2011 where the opposite was seen. Type II was seen in multiple isolates throughout the study period and sporadic cases of types V and VI were noted from 2011 onwards. Only two cases of type I were seen in 2014 and 2015. A number of unknown types was noted from 2010 to 2017. We subsequently further investigated a proportion (*n* = 52) of the unknown types from 2013 to 2016 and found that the majority of the isolates were interpreted as type I-like, type II-like and type III-like [30].

**Fig. 2:**
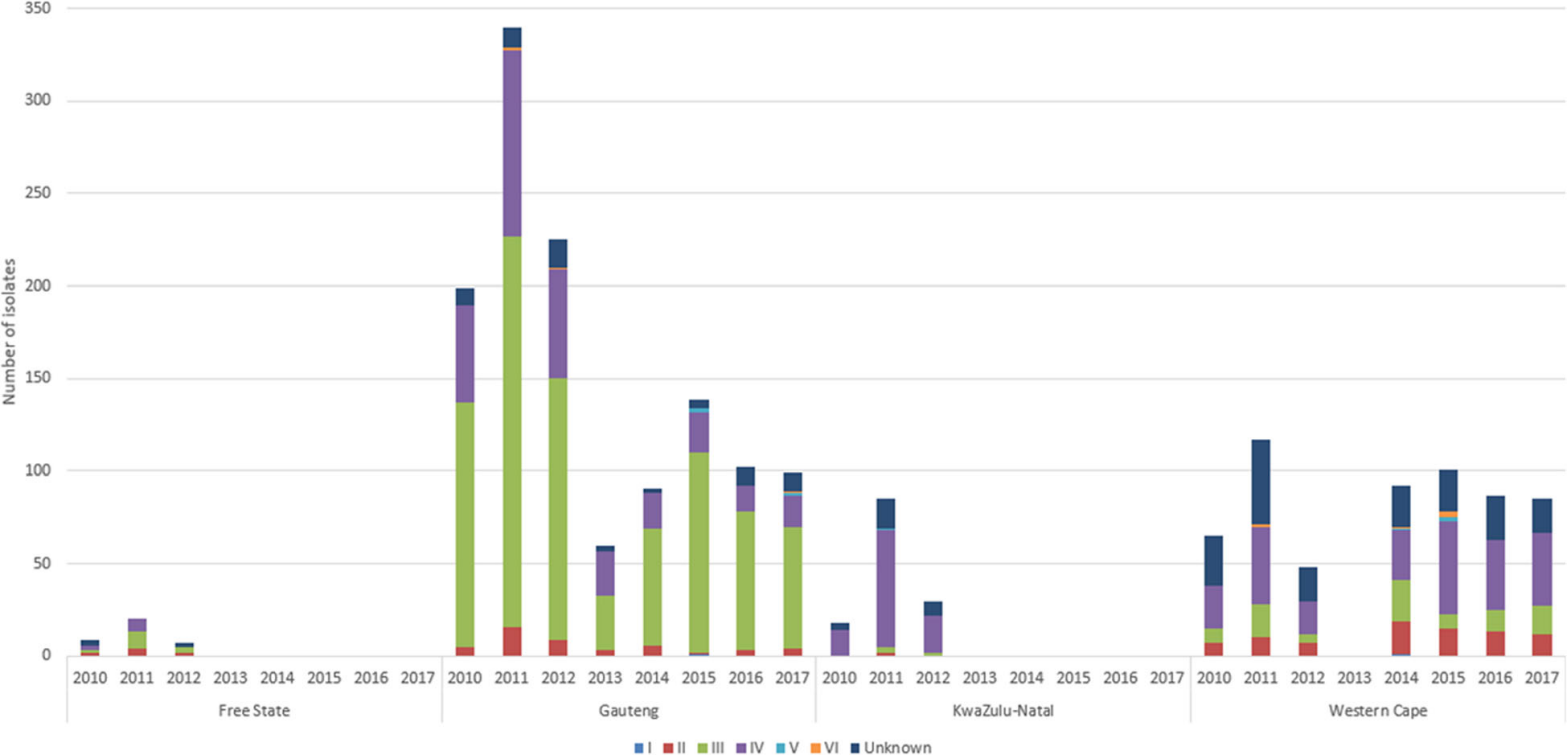
Distribution of SCC*mec* types per province per year in *mec*A-positive isolates (*n* = 2019)

The distribution of SCC*mec* types per province per year is seen in Fig. 2. Type IV predominated in KwaZulu-Natal whereas type III predominated in the remaining three provinces. All six SCC*mec* types including unknown types were observed in Gauteng and the Western Cape provinces.

Antibiotic non-susceptible phenotypes were examined and the distribution of SCC*mec* types per non-susceptible phenotype is seen in Table 1. Isolates belonging to all SCC*mec* types were resistant to most of the antibiotics with the exception of type I. All isolates were susceptible to vancomycin. Type III predominated in azithromycin-, erythromycin-, oxacillin-, cefoxitin-, penicillin-, trimethoprim/sulfamethoxazole-, daptomycin-, tetracycline-, ciprofloxacin-, levofloxacin-, moxyfloxacin- and gentamicin-non-susceptible isolates. Type II predominated in clindamycin-non-susceptible isolates and type IV predominated in rifampicin-non-susceptible isolates.

**Table 1:** Distribution of SCC*mec* types according to antibiotic non-susceptibility phenotypes for MRSA isolates (*n* = 2019)

Antibiotic non-susceptibility phenotype	SCC***mec*** type							
	I	II	III	IV	V	VI	Untypeable	Negative
**Erythromycin**	0	133 (6.58%)	920 (45.56%)	391 (19.36%)	4 (0.19%)	2 (0.09%)	249 (12.33%)	2 (0.09%)
**Clindamycin**	1 (0.04%)	129 (6.38%)	106 (5.25%)	31 (1.53%)	0	4 (0.19%)	30 (1.48%)	0
**Oxacillin**	2 (0.09%)	135 (6.68%)	916 (45.36%)	648 (32.09%)	7 (0.34%)	9 (0.44%)	271 (13.42%)	2 (0.09%)
**Penicillin**	2 (0.09%)	138 (6.83%)	926 (45.86%)	651 (32.24%)	7 (0.34%)	9 (0.44%)	274 (13.57%)	2 (0.09%)
**Trimethoprim/Sulfamethoxazole**	0	12 (0.59%)	883 (43.73%)	542 (26.84%)	2 (0.09%)	3 (0.14%)	42 (2.08%)	2 (0.09%)
**Daptomycin**	0	0	7 (0.34%)	2 (0.09%)	0	0	2 (0.09%)	0
**Linezolid**	0	3 (0.14%)	3 (0.14%)	3 (0.14%)	0	0	3 (0.14%)	0
**Tetracycline**	0	14 (0.69%)	913 (45.22%)	573 (28.38%)	3 (0.14%)	2 (0.09%)	79 (3.91%)	2 (0.09%)
**Rifampin**	0	14 (0.69%)	62 (3.07%)	571 (28.28%)	1 (0.04%)	2 (0.09%)	19 (0.94%)	2 (0.09%)
**Ciprofloxacin**	0	134 (6.63%)	917 (45.41%)	589 (29.17%)	4 (0.19%)	3 (0.14%)	76 (3.76%)	2 (0.09%)
**Levofloxacin**	0	134 (6.63%)	916 (45.36%)	512 (25.35%)	3 (0.14%)	3 (0.14%)	75 (3.71%)	2 (0.09%)
**Moxyfloxacin**	0	133 (6.58%)	918 (45.46%)	510 (25.26%)	3 (0.14%)	2 (0.09%)	75 (3.71%)	2 (0.09%)
**Gentamicin**	0	19 (0.94%)	912 (45.17%)	556 (27.53%)	6 (0.29%)	3 (0.14%)	242 (11.98%)	2 (0.09%)

Susceptibility was classified according to CLSI guidelines [26]

Suggested antibiotics approved by the US Food and Drug Administration (FDA) for clinical use were included in the table. Antibiotics excluded were azithromycin as erythromycin is a surrogate for macrolides, cefoxtin as oxacillin is included for MRSA, those that are recommended for urine only as well as those that were not tested for using the MicroScan Gram-positive PM-33 panel. In addition, vancomycin was excluded as all isolates were susceptible

Majority of the isolates cultured were from adult patients (959/2019, 47.5%). Isolates from paediatric patients were represented by 44.8% (904/2019); the data for the remaining isolates (156/2019, 7.7%) was unknown. The predominating SCC*mec* type in isolates from adults was type IV (478/2019, 23.7%) followed by type III (265/2019, 13.1%), II (123/2019, 6.1%), unknown type (81/2019, 4.0%), V (4/2019, 0.2%) and VI (8/2019, 0.4%). Type I was not seen in isolates cultured from adult patients. The predominating SCC*mec* type in isolates from paediatric patients was type III (569/2019, 28.2%) followed by unknown types (188/2019, 9.3%), type IV (129/2019, 6.4%), II (13/2019, 0.6%), V (3/2019, 0.1%) and I (2/2019, 0.1%). Type VI was not seen in isolates cultured from paediatric patients. The predominating SCC*mec* types in isolates obtained from male and female patients were very similar. The predominating SCC*mec* type could not be correctly established from isolates obtained from patients that died versus those that recovered or were discharged due to the majority of cases having unknown data. The same is applicable for diagnosis.

### *Spa* typing

*Spa* typing was performed on 1467 isolates; the remaining 552 isolates from the period 2010 to 2012 do not have *spa* types assigned. A total of 71 different *spa* types and 24 novel *spa* types were observed. Five isolates were untypable even upon repeat processing. Table 2 shows the distribution of predominating *spa* types over the seven and a half-year period. *Spa* type t037 was the most common and predominated throughout followed by t1257. *Spa* types t012, t045 and t064 were also constantly present over this time period. *Spa* type t4864 was seen only in 2014, t1467 was seen only in 2015, t718 was seen only in 2016 and t5691 emerged in 2017. The remaining *spa* types were seen in small numbers and not consistently throughout the seven and a half-year period.

**Table 2:** Distribution of predominating spa types per year

***Spa*** type	2010	2011	2012	2013	2014	2015	2016	2017
**t012**	11	11	1	3	18	12	14	14
**t037**	136	116	53	28	82	114	85	83
**t045**	31	10	4	1	17	27	22	19
**t064**	18	15	2	4	3	3	2	2
**t1257**	52	57	12	15	29	43	28	33
**t022**	0	1	1	2	0	0	0	1
**t118**	0	1	1	2	0	0	0	0
**t018**	1	1	1	0	5	0	1	0
**t032**	5	0	0	0	2	6	7	4
**t1971**	0	0	1	1	1	4	7	7
**t1443**	5	4	0	0	1	2	0	0
**t1467**	0	0	0	0	0	5	0	0
**t1476**	0	1	0	0	1	3	6	1
**t5691**	0	0	0	0	0	0	0	4
**t021**	0	0	1	1	0	1	0	2
**t148**	3	1	0	0	0	0	1	0
**t238**	3	0	0	0	1	0	0	0
**t294**	0	0	0	0	1	2	0	1
**t451**	1	2	0	0	0	0	0	0
**t718**	0	0	0	0	0	0	2	0
**t891**	1	1	0	0	2	0	1	0
**t4833**	1	2	1	0	0	0	0	0
**t4864**	0	0	0	0	2	0	0	0
**t2029**	1	3	0	0	0	0	0	0

*Spa* types not represented in the table are those that represent only a single isolate per year for all years

Table 3 shows the variation of *spa* types over the seven and a half-year period. The most number of *spa* types were seen in 2011 and the most number of novel *spa* types occurred in 2014, which also showed a high variation in the number of different *spa* types observed. No novel *spa* types were found in 2013.

**Table 3:** Variation of *spa* types per year

Year	No. of different spa types	No. of novel spa types
2010	18	2
2011	29	7
2012	15	4
2013	11	0
2014	21	10
2015	23	6
2016	19	5
2017	22	2

The Gauteng province showed the most variation with 44 different *spa* types and 14 novel *spa* types followed by the Western Cape (*n* = 40 and *n* = 14), respectively. In KwaZulu-Natal 12 different *spa* types were seen and in the Free State eight different *spa* types were observed. One novel *spa* type was found in both KwaZulu-Natal and the Free State provinces but these *spa* types differed from each other. Only t012, t045, t064 and t1257 were observed in all four provinces; t037 was seen in all provinces except in KwaZulu-Natal and t1971 was seen in all provinces except in the Free State; t9061 was seen only in the Free State and t13165, t1555, t4268 and t951 were seen only in KwaZulu-Natal. Two *spa* types (t209 and t2293) were found in the Gauteng and Free State provinces, which also had one novel *spa* type. Three *spa* types (t148, t451 and t891) were found in the Gauteng and KwaZulu-Natal, which also had one novel *spa* type. Nine *spa* types (t008, t018, t021, t022, t032, t1443, t1476, t304, t718) and two novel *spa* types were observed in Gauteng and the Western Cape provinces. Twenty-four different *spa* types (t10304, t105, t1096, t1107, t118, t127, t174, t186, t1943, t272, t2724, t355, t421, t4410, t463, t4833, t4864, t5961, t701, t729, t7962, t840, t913 and t932) and 10 novel *spa* types were seen in Gauteng alone. Twenty-two different *spa* types (t015, t0121, t0379, t059, t11775, t1467, t1774, t1813, t223, t230, t238, t2409, t2526, t294, t324, t432, t498, t5483, t578, t6330, t6931 and t8636) and 10 novel *spa* types were seen in the Western Cape alone.

Antibiotic non-susceptible phenotypes were examined and the distribution of *spa* types representing majority of the isolates is seen in Table 4. One isolate belonging to *spa* type t10304 was non-susceptible to penicillin only (data not shown in table). All three isolates typed as t0379 displayed the same phenotypic profile and were non-susceptible to the fluoroquinolones and beta-lactam antibiotics only (data not shown in table). All four isolates typed as t2029 showed resistance to all antibiotics listed except for daptomycin, linezolid and rifampin (data not shown in table). All four isolates belonging to type t238 and t294 showed the same phenotypic profile and two isolates belonging to t304 and t421 displayed the same phenotypic profile (data not shown in table).

**Table 4:** Distribution of *Spa* types according to antibiotic non-susceptibility phenotypes for MRSA isolates (*n* = 1467)

***Spa*** type	Antibiotic non-susceptibility phenotype														
	Azithromycin	Erythromycin	Clindamycin	Oxacillin	Cefoxitin	Penicillin	Trimethoprim/ Sulfamethoxazole	Daptomycin	Linezolid	Tetracycline	Rifampin	Ciprofloxacin	Levofloxacin	Moxyfloxacin	Gentamicin
t012 (*n* = 84)	28 (1.90%)	81 (5.52%)	71 (4.83%)	84 (5.72%)	84 (5.72%)	84 (5.72%)	11 (0.74%)	0	4 (0.27%)	14 (0.95%)	7 (0.47%)	84 (5.72%)	84 (5.72%)	83 (5.65%)	17 (1.15%)
t018 (*n* = 9)	4 (0.27%)	8 (0.54%)	7 (0.47%)	8 (0.54%)	9 (0.61%)	9 (0.61%)	0	0	0	1 (0.06%)	1 (0.06%)	9 (0.61%)	9 (0.61%)	9 (0.61%)	2 (0.13%)
t032 (*n* = 24)	4 (0.27%)	12 (0.81%)	3 (0.20)	24 (1.63%)	24 (1.63%)	24 (1.63%)	1 (0.06%)	0	0	2 (0.13%)	43 (2.93%)	23 (1.56%)	23 (1.56%)	23 (1.56%)	3 (0.20)
t037 (*n* = 697)	327 (22.29%)	690 (47.03%)	81 (5.52%)	693 (47.23%)	695 (47.37%)	698 (47.58%)	659 (44.92%)	5 (0.34%)	1 (0.06%)	679 (46.28%)	0	691 (47.10)	688 (46.89%)	690 (47.03%)	681 (46.42%)
t045 (*n* = 131)	53 (3.6%)	130 (8.86%)	13 (0.88%)	130 (8.86%)	130 (8.86%)	130 (8.86%)	11 (0.74%)	2 (0.13%)	4 (0.27%)	18 (1.22%)	6 (0.40%)	25 (1.70%)	24 (1.63%)	26 (1.77%)	123 (8.38%)
t064 (*n* = 49)	21 (1.43%)	29 (1.97%)	0	49 (3.34%)	49 (3.34%)	49 (3.34%)	44 (2.99%)	0	0	47 (3.20%)	47 (3.20%)	30 (2.04%)	25 (1.70_	26 (1.77%)	47 (3.20%)
t1257 (*n* = 269)	79 (5.3%)	164 (11.17)	14 (0.95%)	268 (18.26%)	268 (18.26%)	268 (18.26%)	249 (16.97%)	0	2 (0.13%)	263 (17.92)	255 (17.38%)	264 (17.99%)	231 (15.74%)	231 (15.74%)	252 (17.17%)
t1443 (*n* = 14)	1 (0.06%)	1 (0.06%)	0	14 (0.95%)	13 (0.88%)	14 (0.95%)	13 (0.88%)	0	0	13 (0.88%)	14 (0.95%)	13 (0.88%)	13 (0.88%)	13 (0.88%)	13 (0.88%)
t1476 (*n* = 12)	0	5	0	12 (0.81%)	12 (0.81%)	12 (0.81%)	2 (0.13%)	0	0	9 (0.61%)	0	10 (0.68%)	7 (0.47%)	7 (0.47%)	11 (0.74%)
t1971 (*n* = 23)	2 (0.13%)	19 (1.29%)	1 (0.06%)	23 (1.56%)	23 (1.56%)	23 (1.56%)	23 (1.56%)	0	0	23 (1.56%)	23 (1.56%)	23 (1.56%)	21 (1.42%)	21 (1.42%)	23 (1.56%)
Novel *spa* types (*n* = 38)	9 (0.61%)	24 (1.63%)	7 (0.47%)	37 (2.52%)	37 (2.52%)	38 (2.59%)	23 (1.56%)	0	0	25 (1.70%)	16 (1.09%)	31 (2.11%)	29 (1.97%)	27 (1.84%)	27 (1.84%)
Untypeable (*n* = 5)	3 (0.20%)	4 (0.27%)	1 (0.06%)	5 (0.34%)	5 (0.34%)	5 (0.34%)	3 (0.20%)	0	0	3 (0.20%)	1 (0.06%)	4 (0.27%)	4 (0.27%)	3 (0.20%)	3 (0.20%)

The following *spa* types were excluded from the table as they accounted for a small number of isolates: t008, t0121, t015, t021, t022, t0379, t059, t10304, t105, t1096, t1107, t11775, t118, t127, t13165, t1467, t148, t1555, t174, t1774, t1813, t186, t1994, t2029, t223, t2293, t230, t238, t2409, t2526, t272, t2724, t294, t304, t324, t355, t379, t421, t4268, t432, t4410, t451, t463, t4833, t4864, t498, t5483, t5691, t578, t6330, t6931, t701, t718, t729, t7962, t840, t8636, t891, t9061, t913, t932, t951

Susceptibility was classified according to CLSI guidelines [26]

Suggested antibiotics approved by the US Food and Drug Administration (FDA) for clinical use were included in the table. Antibiotics excluded were those that are recommended for urine only as well as those that were not tested for using the MicroScan Gram-positive PM-33 panel. In addition, vancomycin was excluded as all isolates were susceptible

A total of 552 isolates were not typed

Of the known adult vs paediatric information, the predominating *spa* type in isolates from adults was t1257 (195/1467, 13.3%) followed by t037 (189/1467, 12.7%), t012 (70/1467, 4.8%), t064 (32/1467, 2.2%), t1971 (20/1467, 1.4%), t032 (19/1467, 1.3%) and t045 (15/1467, 1%). The remaining *spa* types within this group individually represented less than 1%. This group consisted of 55 different *spa* types and 18 novel *spa* types. Two isolates were untypeable. The predominating *spa* type in isolates from paediatric patients was t037 (446/1467, 30.4%) followed by t045 (115/1467, 7.8%) and t1257 (53/1467, 3.6%). The remaining *spa* types within this group individually represented less than 1%. This group consisted of 32 different *spa* types and 10 novel *spa* types. Three isolates were untypeable. The following *spa* types were seen in isolates from adult patients only: t008, t0121, t018, t021, t0379, t059, t1175, t118, t1467, t174, t1774, t1813, t2029, t223, t2293, t230, t2409, t2526, t294, t304, t324, t379, t432, t4410, t463, t4864, t578, t6931, t701, t729, t7962, t840, t8636, t9061 and t913. There were 14 novel *spa* types in this group. The following *spa* types were seen in isolates from paediatric patients only: t10304, t1096, t127, t13165, t1555, t186, t1943, t272, t355, t4286, t498, t5483, t6330 and t932; six novel spa types were observed in this group. The predominating *spa* types in isolates obtained from male and female patients were very similar. Furthermore, the predominating *spa* type could not be correctly established from isolates obtained from patients that died versus those that recovered or were discharged due to the majority of cases having unknown data. The same is applicable for diagnosis.

### SCC*mec* and *spa* types complexes

The SCC*mec*-*spa* type combinations are referred to as complexes. A total of 1467 SCC*mec*-*spa* type complexs were obtained. The five isolates that were not typeable for *spa* type were excluded from the analysis; SCC*mec* types for each of these varied (SCC*mec* II, III, IV, V and unknown type). The most diverse complex was composed of the SCC*mec* type IV element and 53 different *spa* types. Next were the isolates with unknown SCC*mec* type; these were associated with 28 different *spa* types. SCC*mec* type III was associated with 24 different *spa* types and SCC*mec* type II was associated with 20 different *spa* types. There were smaller numbers of SCC*mec* type I, V and VI isolates and predominance was therefore inconsequential; the isolates varied with regard to *spa* type. The SCC*mec*-*spa* type combinations constituting the complexes are shown in Table 5.

**Table 5:** SCC*mec*- *spa* type combinations

SCC***mec*** type, n	***Spa*** type, n (%)
SCC*mec* type I isolates (*n* = 2)	t015, t186 (*n* = 1, 50%, each).
SCC*mec* type II (*n* = 104)	t012 (*n* = 71, 67.6%); t037 (*n* = 7, 6.7%); t021 (*n* = 4, 3.8%); t238 (*n* = 4, 3.8%); t1257 (*n* = 3, 2.9%); t018 (*n* = 2, 1.9%); t0121 (*n* = 1, 0.9%); t045 (*n* = 1, 0.9%); t064 (*n* = 1, 0.9%); t2526 (*n* = 1, 0.9%); t4864 (*n* = 1, 0.9%); t6330 (*n* = 1, 0.9%); t729 (*n* = 1, 0.9%); t840 (*n* = 1, 0.9%); t8636 (*n* = 1, 0.9%); t913 (*n* = 1, 0.9%); novel *spa* types: txAF, txAK, txAO, txAQ (*n* = 1, 0.9%, each).
SCC*mec* type III (*n* = 709)	t037 (*n* = 656, 92.5%); t045 (*n* = 12, 1.7%); t1257 (*n* = 8, 1.1%); t012 (*n* = 8, 1.1%); t2029 (*n* = 4, 0.6%); t0421 (*n* = 2, 0.3%); t1476 (*n* = 2, 0.3%); t032 (*n* = 1, 0.1%); t127 (*n* = 1, 0.1%); t2293 (*n* = 1, 0.1%); t355 (*n* = 1, 0.1%); t932 (*n* = 1, 0.1%); t7962 (*n* = 1, 0.1%); t701 (*n* = 1, 0.1%); t1943 (*n* = 1, 0.1%); t4410 (*n* = 1, 0.1%); t5691 (*n* = 1, 0.1%); novel *spa* types: txAB, txAF, txAM, txAN, txAP, txAZ, txBD (*n* = 1, 0.1%, each).
SCC*mec* type IV (*n* = 451)	IV-t1257 (*n* = 255, 56.5%); t064 (*n* = 47, 10.4%); t1973 (*n* = 23, 5.1%), t032 (*n* = 21, 4,7%); t1443 (*n* = 14, 3.1%); t037 (*n* = 12, 2.7%); t022 (*n* = 5, 1.1%); t1467 (*n* = 5, 1.1%), t118 (*n* = 4, 0.9%,); t294 (*n* = 4, 0.9%,); t4833 (*n* = 4, 0.9%); t451 (*n* = 3, 0.7%); t891 (*n* = 3, 0.7%); t012 (*n* = 2, 0.4%); t105 (*n* = 2, 0.4%); t2293 (*n* = 2, 0.4%); t304 (*n* = 2, 0.4%); t718 (*n* = 2, 0.4%); t008 (*n* = 1, 0.2%); t015 (*n* = 1, 0.2%); t018 (*n* = 1, 0.2%); t0379 (*n* = 1, 0.2%); t045 (*n* = 1, 0.2%); t059 (*n* = 1, 0.2%); t1555 (*n* = 1, 0.2%); t1774 (*n* = 1, 0.2%); t230 (*n* = 1, 0.2%); t272 (*n* = 1, 0.2%); t2724 (*n* = 1, 0.2%); t324 (*n* = 1, 0.2%); t379 (*n* = 1, 0.2%); t4268 (*n* = 1, 0.2%); t432 (*n* = 1, 0.2%); t4864 (*n* = 1, 0.2%); t5691 (*n* = 1, 0.2%); t578 (*n* = 1, 0.2%); t951 (*n* = 1, 0.2%); novel *spa* types: txAA (*n* = 5, 1.1%), txAC and txAI (*n* = 2, 0.4%), each), txAD, txAE, txAG, txAJ, txAL, txAM, txAN, txAS, txAO, txBA, txBB, txBC txBE (*n* = 1, 0.2%, each).
SCC*mec* type V (*n* = 3)	t1476, t045, t037 (*n* = 1, 33.3%, each)
SCC*mec* type VI (*n* = 5)	t1813 (*n* = 2, 80%); t174 (*n* = 1, 20%); t223 (*n* = 1, 20%); novel *spa* type txAD (*n* = 1, 20%).
Unknown SCC*mec* types (*n* = 190)	t045 (*n* = 114, 60%); t037 (*n* = 22, 11.6%); t1476 (*n* = 9, 4.7%); t018 (*n* = 6, 3.2%); t148 (*n* = 5, 2.6%); t012 (*n* = 3, 1.6%); t1257 (*n* = 3, 1.6%); t13165 (*n* = 3, 1.6%);, t008 (*n* = 2, 1.1%,); t032 (*n* = 2, 1.1%,); t5691 (*n* = 2, 1.1%,); t891 (*n* = 2, 1.1%,); t021 (*n* = 1, 0.5%); t064 (*n* = 1, 0.5%); t10304 (*n* = 1, 0.5%); t1096 (*n* = 1, 0.5%); t1107 (*n* = 1, 0.5%); t11775 (*n* = 1, 0.5%); t2409 (*n* = 1, 0.5%); t463 (*n* = 1, 0.5%); t498 (*n* = 1, 0.5%); t5483 (*n* = 1, 0.5%); t6931 (*n* = 1, 0.5%); t9061 (*n* = 1, 0.5%); novel *spa* types: txAH (*n* = 2, 1.1%), txAD (*n* = 1, 0.5%), txAF (*n* = 1, 0.5%), txAL (*n* = 1, 0.5%).

### Predominating circulating clones

MRSA clones were defined by their sequence type (ST), SCC*mec* type and *spa* type. Multilocus Sequence Typing was performed on 48 isolates only. Isolates were selected randomly based on the most common *spa*-types (t037, *n* = 9; t1257, *n* = 10; t012, *n* = 9; t064, *n* = 9; t045, *n* = 8; t032, *n* = 3). The predominating circulating clones based on common *spa* types are seen in Table 6. Although only data for 48 isolates are present, the circulating clones are relatively diverse. As MLST was only done on a few selected isolates we could not confidently establish the circulating clones that are representative of entire surveillance population. We can therefore not comment on the evolution of MRSA clones in our setting.

**Table 6:** Predominating circulating clones

ST (CC)	SCC***mec*** type	***Spa*** type	No. of isolates
5 (5)	III	t045	1
5 (5)	V	t045	2
5 (5)	Unknown	t045	4
5 (5)	Unknown	t1257	1
22 (22)	IV	t012	1
22 (22)	IV	t032	2
4121 (22)	IV	t032	1
36 (30)	II	t012	5
36 (30)	II	t037	1
36 (30)	II	t064	1
36 (30)	III	t045	1
239 (8)	II	t1257	1
239 (8)	III	t1257	1
239 (8)	III	t012	1
239 (8)	III	t037	6
239 (8)	IV	t037	1
239 (8)	Unknown	t012	1
239 (8)	Unknown	t037	1
612 (8)	III	t012	1
612 (8)	IV	t064	8
612 (8)	IV	t1257	6
Unknown^a^	IV	t1257	1

^a^ ST is unknown due to new allele for the *pta* gene; at position 277 the nucleotide adenine (A) is present and not the expected nucleotide, guanine (G)

## Discussion

This study is a detailed description of the molecular characterisation of MRSA isolates with specific focus on SCC*mec* types and *spa* types and, to a lesser extent, sequence types. It is important to have a genetic understanding of the circulating strains in a geographical region to establish genetic diversity, transmission, dissemination, epidemiology and evolution. Antimicrobial susceptibility profiles were also reported; apart from using antimicrobial susceptibility results for treatment regimens, antimicrobial susceptibility profiles are also important in identifying a link to specific genotypes, which could potentially identify virulence patterns. Antimicrobial selection may potentially also be a key factor in the dissemination of predominating MRSA clones within a hospital environment [31].

SCC*mec* type III was the most predominant SCC*mec* type followed by type IV. Type III was also the most frequent SCC*mec* type in studies in Iran [32, 33], Serbia [34], Brazil [35] and Europe [36]. The most prevalent *spa* type in our study was t037. This is in keeping with a review conducted in 2018 of European, Asian, American, Australian and African studies from 2007 onwards including 18 studies from Africa which showed that the most prevalent *spa* type was t037 [5]. The review also showed that t084 and t064 were common in Africa. In contrast to our study, t064 was present in a small number of isolates (*n* = 49) and t084 was not observed at all. Interestingly this review also showed that the most prevalent *spa* type in America was t008, which was reported only in America and Canada. Our current study has shown the occurrence of t008 in three isolates from Gauteng and the Western Cape provinces.

Isolates harbouring SCC*mec* type III and IV elements were the most resistant as evidenced by the large number of non-susceptible phenotypes to majority of the antimicrobial agents (Table 1). A 2014 study in Iran showed similar findings; they further molecularly characterised resistance genes and found that their type III isolates contained different resistance genes [37]. In contrast, an Indian study in 2016 showed more phenotypic resistance to non-beta-lactam antibiotics in their type I isolates [38].

A 2017 Chinese study on 120 MRSA isolates showed differences to the current study; 100 % of their *spa* type t037 isolates were resistant to clindamycin, erythromycin, ciprofloxacin, gentamicin, tetracycline and trimethoprim/sulfamethoxazole whereas only 6% of our isolates were resistant to clindamycin and 45 to 47% were resistant to the remaining antibiotics. However, in keeping with the study from China, none of our t037 isolates were resistant to rifampin and vancomycin (Table 4) [39]. Another Chinese study with 106 t037 isolates showed predominant resistance to clindamycin, erythromycin, ciprofloxacin, gentamicin, tetracycline, trimethoprim/sulfamethoxazole and chloramphenicol [40]. Of six Nigerian t037 isolates, all were resistant to clindamycin, erythromycin, ciprofloxacin, gentamicin, tetracycline and trimethoprim/sulfamethoxazole in addition to penicillin, oxacillin and moxifloxacin [41]; in the current study, almost 50% (47–48%) of the t037 isolates were resistant to penicillin, oxacillin and moxifloxacin.

The study of circulating clones and clonal evolution is important because it is used to assess the relationship between clonal types, disease symptoms, antibiotic choice and clinical outcomes [42]. Clones are bacterial strains that have descended from a common ancestor and through point mutations, recombination, acquisition and deletion of mobile genetic elements they diversify resulting in wide-ranging genotypes and phenotypes [43]. In order to establish circulating clones and clonal evolution, multiple molecular tools should be employed; the combination of ST, SCC*mec* type and *spa* type would ideally be preferred. However, as MLST is more costly, we were not able to perform this technique on all isolates. Studies have shown that SCC*mec* typing is not a very discriminatory method and that *spa* typing alone was not able to clearly predict ST or PFGE type but when combined with BURP analysis producing *spa* CCs, it is sufficient for describing the clonal structure of *S. aureus* [6, 10]. Although useful, it should be noted that *spa* typing takes only one gene into consideration in relation to the entire genome and therefore does not reflect mutational events occurring throughout the genome [5]. Nevertheless, *spa* typing is extremely useful and we have coupled it with SCC*mec* typing and sequence typing to a lesser extent, to provide information on the circulating *S. aureus* strains in our population.

A review manuscript by Asadollahi *et. al*., in 2018 [5] showed that from five African studies, t037 was most associated with SCC*mec* type III (106 isolates) and least associated with type V (one isolate). Our study showed similar findings; t037 was mostly associated with SCC*mec* type III (656 isolates) and least associated with type V (one isolate). In another study of German, French, Japanese and Finnish isolates in 2007, majority of the t037 isolates (*n* = 8) were also associated with SCC*mec* type III [44]. This was also seen in seven isolates from a 2014 Iranian study but two t037 isolates were also associated with SCC*mec* type IV and one was associated with SCC*mec* type I [37].

The Asadollahi *et. al*., review manuscript further showed that t037 was associated with ST239 and t064 was associated with ST8 [5]. In our study, t037 was mainly associated with ST239 but one isolate was associated with ST36. The isolates belonging to t064 were mainly associated with ST612 and one isolate was associated with ST36. The review further showed that t032 was always associated with ST22 irrespective of the continent in which it was observed; one of the t032 isolates in our study also showed this finding whereas the second t032 isolate was associated with ST4122. Both ST22 and ST4121 belong to MLST CC22. As MLST was only performed on a few selected isolates, the results could have potentially differed if ST data was available for more isolates.

Other publications have used ST and the SCC*mec* element to define clonal types [45, 46]. In the current study, the Brazilian/Hungarian clone (ST239-MRSA-III) accounted for eight out of the 48 (17%) isolates typed. This is also a common MRSA strain in New Zealand, where the most common associated *spa* type is t037. Alternative clone names include EMRSA-1, EMRSA-4, EMRSA-11, Por/Bra, Vienna, AUS-2 EMRSA and AUS-3 EMRSA) (http://esr.cri.nz/assets/HEALTH-CONTENT/Images-and-PDFs/MRSAdescriptions.pdf), [45]. Of the eight isolates in the current study, six were *spa* type t037. This clone has also been observed in Finland, Germany, Greece, Ireland, Netherlands, Poland, Portugal, Slovenia, Sweden, United Kingdom and the United States of America [45]. Another common MRSA strain in New Zealand is ST22-MRSA-IV (EMRSA-15, Barnim) (http://esr.cri.nz/assets/HEALTH-CONTENT/Images-and-PDFs/MRSAdescriptions.pdf), most associated with *spa* types t032, t1401 and t5501. In our study, two of the three isolates were t032 and the remaining one being t012. This clone has also been seen in Germany Ireland, Sweden and the United Kingdom [45]. Strain ST-36-MRSA-II (EMRSA-16) also common in New Zealand and most associated with t018 (http://esr.cri.nz/assets/HEALTH-CONTENT/Images-and-PDFs/MRSAdescriptions.pdf) was also seen in seven isolates in the current study; however none were associated with *spa* type t018, five were t012 and one each for t037 and t064. This clone was also seen in Finland and the United Kingdom [45]. Another clonal type observed in our study included ST5-MRSA-III (*n* = 1) which is a Belgian clone [45].

As *spa* typing was not done on all MRSA isolates and as MLST was only performed on a few selected isolates we could not confidently establish the circulating clones that are representative of entire surveillance population. We therefore cannot comment on the evolution of MRSA clones in our setting. However, although ST data was available for 48 isolates only (which also had *spa* and SCC*mec* type data), the circulating clones are relatively diverse and if the ST was omitted and only SCC*mec* and *spa* types considered, the diversity of the circulating strains increases. Nevertheless, of the 48 clones we have observed taking ST, SCC*mec* type and *spa* type into consideration, the most common were ST612-IV-t064 (*n* = 8), ST612-IV-t1257 (*n* = 6), ST239-III-t037 (*n* = 6) and ST36-II-t012. Multiple introductions of ST612 was observed in Western Australia in both human and equine reservoirs [47]. ST612 was also recently observed in the clone ST612-CC8-t1257-SCCmec_IVd(2B) obtained from the poultry food chain in South Africa [48]. In addition to the studies mentioned above, the Brazilian/Hungarian clone ST239-III-t037 was commonly found over a 15 year period in a study in China beginning in 1994 [40]. The presence of this clone was also observed in various continents [49]. Therefore, this clone is very well established globally.

It has been shown that the transformation from a MRSA clone to a MSSA clone can occur through the excision of the SCC*mec* element and consequently the loss of methicillin resistance. Therefore, it is possible for a clone to evolve from MSSA into MRSA through the acquisition of the SCC*mec* element or from MRSA to MSSA through the excision of the SCC*mec* element [50]. Molecular typing is extremely useful in studying genetic diversity and a study on a collection of isolates from 19 countries in Europe, the United Kingdom, The United States and Latin America has shown that MRSA and MSSA differ with regards to the diversity of their genetic backgrounds as MSSA has shown to be more diverse [10]. A limitation of the current study is that molecular typing was performed on MRSA isolates only; results for MSSA is therefore lacking and we cannot make any remarks on this matter. To add to genetic diversity, clones responsible for causing HA infections and CA infections may differ and the recombination between HA and CA clones does occur [50]. A detailed investigation taking into consideration aspects like virulence factors such as surface proteins, invasins, biochemical properties, membrane-damaging toxins, exotoxins e.g. Panton-Valentine Leukocidin (PVL), biofilm production, antimicrobial resistance genes and clinical syndromes [42, 43, 50, 51] would be beneficial.

## Conclusion

This study reports a large dataset of isolates collected from various provinces in South Africa from 2010 to 2017. A variety of *spa* types were observed in this study; this is in keeping with other reports showing the presence of multiple *spa* types in the MRSA population. Moreover, data from Africa is not abundant. It is evident that MRSA clones are diverse; they disseminate both rapidly and efficiently and it is important to understand why particular clones dominate in a specific geographical location in order to develop effective strategies to control the spread of *S. aureus* infections.

## Data Availability

The datasets used and/or analysed during the current study are available from the corresponding author on reasonable request. Access to the National Institute for Comminicable Diseases GERMS-SA database cannot be granted due to the presence of patient identifiers.

## Abbreviations

HA: Healthcare-associated
CA: Community-associated
MSSA: Methicillin-susceptible *S. aureus*
MRSA: Methicillin-resistant *S. aureus*
PFGE: Pulsed-field Gel Electrophoresis
MLST: Multilocus Sequence Typing
*spa*: Stapylococcal protein A
SCC*mec*: Staphylococcal Cassette Chromosome *mec*
BURST: Based Upon Related Sequence Type
ST: Sequence type
CC: Clonal complex
BURP: Based Upon Repeat Pattern
VNTR: Variable-number tandem repeat
*ccr*: Cassette chromosome recombinase
DMP: Diagnostic Media Products
NHLS: National Health Laboratory Service
NICD: National Institute for Communicable Diseases
AST: Antimicrobial susceptibility testing
CLSI: Clinical and Laboratory Standards Institute
PCR: Polymerase Chain reaction
